# A scalable suspension insect cell transfection method for production of baculoviruses with low amplification passages

**DOI:** 10.1016/j.mex.2020.101103

**Published:** 2020-10-15

**Authors:** Aline Diniz Cabral, Felipe Baena Garcia, Renata Torres da Costa, Ligia Marinho Pereira Vasconcelos, Mabel Uehara, Edmar Silva Santos, Márcia Aparecida Sperança

**Affiliations:** Centro de Ciências Naturais e Humanas, Universidade Federal do ABC, Campus São Bernardo do Campo, Rua Arcturus, 03 - Jardim Antares, Bloco Delta, Sala 226, Laboratório 107, CEP 09606-070, São Bernardo do Campo, São Paulo, Brazil

**Keywords:** Baculovirus, Insect cell, Sf9, Transfection

## Abstract

Baculovirus expression vector systems (BEVS) have been widely used for production of recombinant proteins in insect cells. However, baculoviruses superinfection and repeated passages originate defective interfering particle (DIP) mutants, which is a limitation to a continuous large-scale production. Accordingly, a classical chemical transfection method performed on monolayer of *Spodoptera frugiperda* insect cells (Sf9) was modified to produce recombinant baculoviruses with high efficiency. Modifications consist to transfect exponentially growing cells in suspension after concentration by tenfold through centrifugation. Ten different constructions of recombinant baculoviruses with insert varying in size from 180 bp to 2,395 bp, were obtained through employment of the *Bac-to-Bac* expression system (ThermoFisher/Invitrogen). The transfection efficiency of the modified protocol varied from 45 to 57%, independent of the insert size, while classical method present transfection efficiency of 2 to 20%. After transfection of 6 × 10^6^ cells, the recombinant baculoviruses titer obtained with modified method was about 2 × 10^7^ pfu/ mL in a total volume of 12 mL, which is scalable to 24 liters of 1 × 10^8^ pfu/ mL, after only two amplification rounds, contributing to improve large scale heterologous protein production in insect cells, with low amplification passages.

## Graphical abstract

**Figure:**
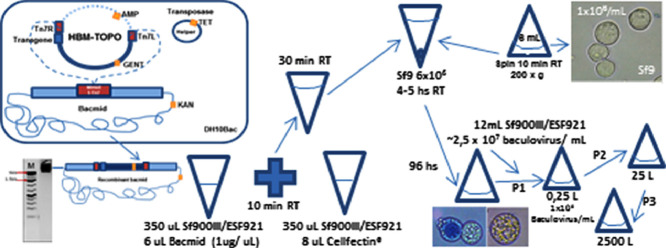

Specifications table

**Table:** 

Subject Area	Biochemistry, Genetics and Molecular Biology
More specific subject area	Transfection of eukaryotic cells
Protocol name	Transfection of insect cells in suspension
Reagents/tools	Bac-to-Bac expression system – Invitrogen/Thermo Fischer; Cellfectin transfection reatent - Invitrogen/Thermo Fischer
Experimental design	Iin this work, a chemical transfection method used to introduce baculovirus DNA into *Spodoptera frugiperda* insect cells (Sf9) grown on monolayer was modified to be used in cells grown in suspension, to produce recombinant baculoviruses with high efficiency. Classical method consist to transfect 6 × 10^6^ cells/ 75 cm^2^ culture flask in monolayer in 12 mL of culture medium containing 6 ug of DNA and 48 uL of Cellfectin^Ⓡ^. In the modified method, 6 × 10^6^ cells of exponentially suspension growing Sf9 cells are concentrated by centrifugation and suspended in 700 uL of transfection solution containing serum-free culture medium, 6 ug of DNA and 8 uL of Cellfectin^Ⓡ^. After incubation for 4–5 h, the transfected cells are centrifuged, suspended in 12 mL of culture medium, and incubated in shaker at 28 °C and 100 rpm for 96 h.
Value of the Protocol	• The Sf9 insect cells grown in suspension were efficiently transfected at a tenfold higher concentration than used in conventional methods, • The transfection method efficiency was independent of the recombinant bacmid insert size, • The obtained results enable large scale production of recombinant proteins using baculoviruses with low amplification passages.

## Background

Baculovirus expression vector systems (BEVS) were first described in 1983 [1], and since then they have been widely used for the production of recombinant proteins in insect cells. Most BEVS are based on the *Autographa californica multicapsid nucleopolyhedrovirus* (AcMNPV) and the expression of recombinant proteins is generally driven by the polyhedrin or p10 strong insect-virus genes promoters. The main insect cell line used in BEVS is called Sf9, and it is derived from the ovarian tissue of *Spodoptera frugiperda* [2]. Spite of all excellent characteristics of BEVS in the production of recombinant proteins, baculoviruses superinfection and repeated passages of virus originate defective interfering particle (DIP) mutants, which is a limitation to continuous large-scale baculovirus production in insect cells [3]. Accordingly, improvement of the initial acquirement of recombinant baculoviruses by transfection could contribute to diminish the number of virus amplification passages to obtain high titers to be used in large scale protein production in insect cells. Thus, in this protocol, the transfection of recombinant baculovirus DNA (Bacmid) in exponentially growing Sf9 cells concentrated by tenfold is presented and resulted in high titer of baculoviruses, through low amplification passages.

## Method details

### Insect cell culture and bacmids construction

**Fig. 1:**
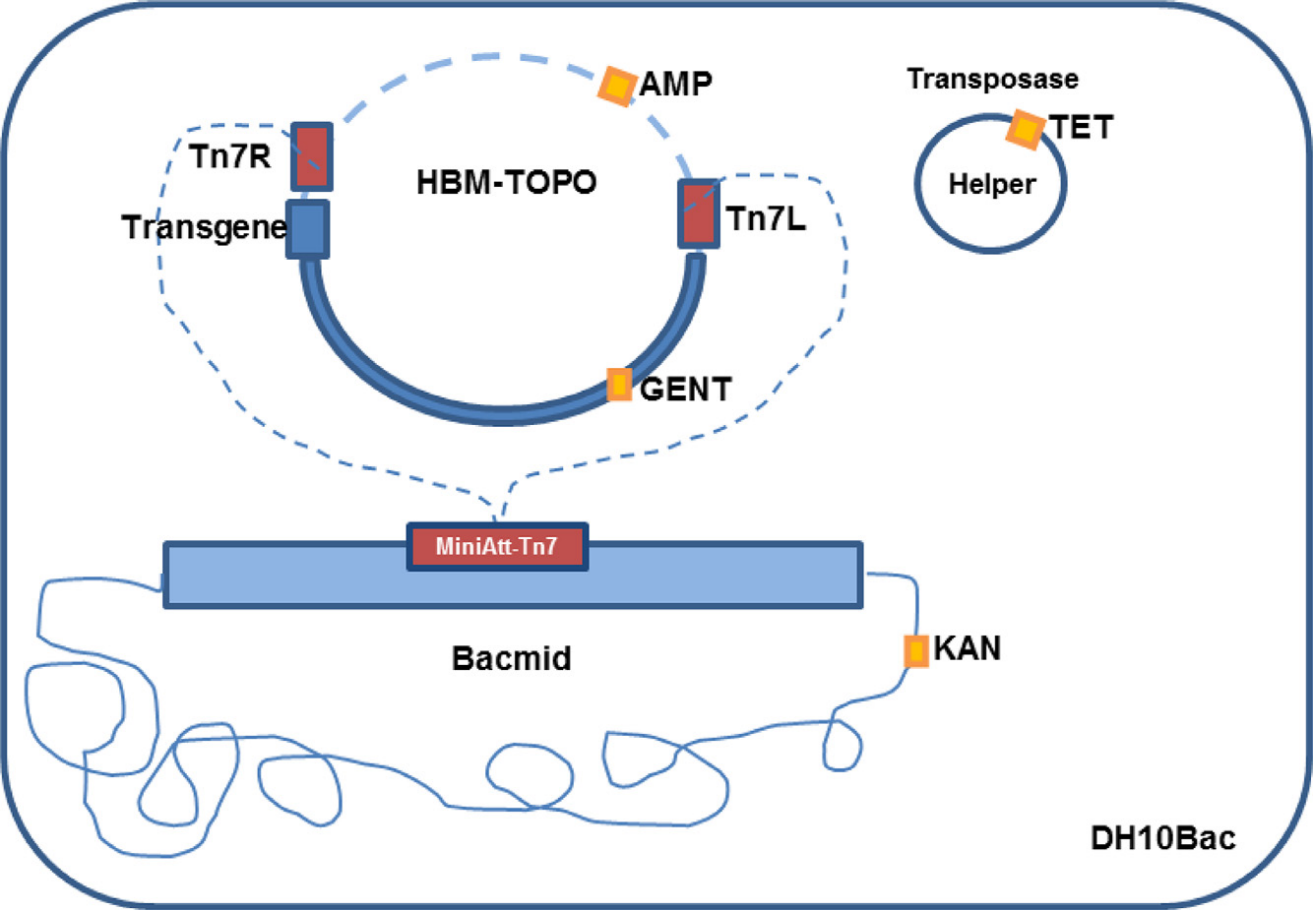
Schematic representation of recombinant bacmid construction through transposition process. Tn7R and Tn7L: transposase recognition sequence, MiniAtt-Tn7: transposase sequence target; TET, GENT, AMP, KAN: tetracycline, gentamycin, ampicillin and kanamycin resistance marker. (Adapted from: Invitrogen - Bac-to-Bac^Ⓡ^ Baculovirus Expression System User Guide, 2013).

With the employment of the BEV system *Bac-to-Bac* HBM-TOPO purchased from Thermo Scientific/Invitrogen, construction of recombinant bacmids were performed for ten different protein encoding genes (Table 1). The *Bac-to-Bac* HBM-TOPO system is based on AcMNPV and the recombinant expression is driven by the Polyhedrin gene promoter associated to a honeybee melitin (HBM) exportation signal at the N-terminus that has the function to transport the product to the extracellular medium. The vector also presents a C-terminal tag of six histidines (his-tag) to facilitate protein purification using affinity chromatography which is preceded by the Tobacco Etch Virus (TEV) protease signal peptide. The encoding sequences described in Table 1 were amplified by PCR with the *Platinum Taq High Fidelity* (ThermoFisher Scientific/Invitrogen) and blunt ended fragments were obtained by treatment of the amplicons with the proofreading *Pfx* Taq DNA polymerase (ThermoFisher Scientific/Invitrogen) for 30 min at 74 °C to remove the adenine 3′OH overhang added by the proofreading *Platinum Taq High Fidelity* DNA polymerase. This procedure was necessary since not all fragments could be obtained directly by PCR with *Pfx* Taq DNA polymerase. Cloning of coding sequences into HBM TOPO donor plasmid and production of recombinant bacmid were performed according to manufacturer instructions, through transposition of the transgenic construction from HBM-TOPO to bacmid in the DH10B *Escherichia coli* strain (Fig. 1).

**Table 1:** Protein encoding genes used to construct recombinant baculoviruses, transfection efficiency and virus titer.

Encoding gene	Amplicon	Transfection efficiency^f^	Virus titer^h^
May^a^ Nsp2	2395 bp	48%	2,4 × 10^7^ virus/mL
May E1	1308 bp	52%	2,6 × 10^7^ virus/mL
May 6kE1	1488 bp	47%	2,3 × 10^7^ virus/mL
May E2	1209 bp	45%	2,2 × 10^7^ virus/mL
May E3	198 bp	55%	2,7 × 10^7^ virus/mL
May 6k	180 bp	57%	2,8 × 10^7^ virus/mL
ChitLamz^b^	1287 bp	52% (50%, 53%, 51%)^g^	2,6 × 10^7^ virus/mL
ChitLbraz^c^	1287 bp	51% (50%, 50%, 53%)^g^	2,5 × 10^7^ virus/mL
ChitLinf^d^	1287 bp	55% (58%, 53%, 54%)^g^	2,7 × 10^7^ virus/mL
ChitLmex^e^	1287 bp	49% (48%, 50%, 49%)^g^	2,4 × 10^7^ virus/mL

**a.***Mayaro* alphavirus isolated in Acrelândia, city localized in the Brazilian State of Acre (Terzian et al., 2015); Nsp2 – non-structural protein 2; 6k, E1, E2, and E3 correspond to structural proteins; **b-e** correspond to chitinase encoding gene from *Leishmania amazonensis,* L. *braziliensis,* L. *infantum* and L. *mexicana*, respectively; **f** – percentage of cells with cytophatic effect 48–72 h post transfection; **g** each chitinase construction was repeated three times and the individual transfection efficiency values are represented in brackets; **h** correspond to virus titer estimative according to transfection efficiency of 6 × 10^6^ cells in a total culture volume of 12 mL.

### Transfection of Sf9 grown in suspension and microscopic analysis

Transfections of Sf9 insect cells were performed employing two different serum free culture medium, the ESF 921 (Expression Systems LLC) and SF900 III (ThermoFisherScientific/Invitrogen). Initially, two microtubes with 350 uL of ESF-921 or SF900III insect cell culture medium containing 8 µL of *Cellfectin*^Ⓡ^ *Reagent* (Invitrogen) and 6 µg (1 µg / µL) of purified recombinant baculovirus DNA, respectively, were incubated for 10 min at room temperature. Afterwards, *Cellfectin*^Ⓡ^ and baculovirus DNA solutions were mixed and incubated for 30 min at room temperature (transfection mixture). In parallel, Sf9 cells were grown in 250 mL polycarbonate Erlenmeyers (Nunc) with a maximum of 100 mL of appropriate insect cell medium, in shaker protected from light, at 100 rpm, 28 °C, until reaching a density of 1 × 10^6^ cell/ mL and presenting more than 95% viability determined after staining with 0,5% of trypan blue. Six milliliters of 1 × 10^6^ cell/ mL were harvested by centrifugation at 200 x g, 10 min, at room temperature. The supernatant was removed and cells were suspended in the transfection mixture and incubated at room temperature for 4 to 5 h. Following incubation, cells were harvested by centrifugation at 200 x g for 15 min at room temperature and after removal of the supernatant they were gently dissolved with 12 mL of ESF 921 or SF900III insect cells culture medium and transferred to a 25 mL sterile Erlenmeyer polycarbonate flask and incubated in shaker at 100 rpm at 28 °C, for 96 h.

**Fig. 2:**
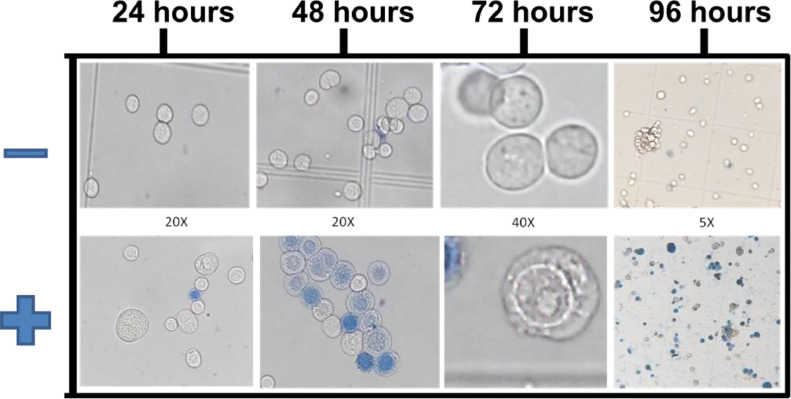
Microscopic analysis of Sf9 insect cells stained with trypan blue after transfection with Mayaro Nsp2 recombinant baculovirus DNA. Non-transfected (-) and transfected (+) Sf9 cells after 24, 48, 72 and 96 h post transfection experiment. Microscopic magnification of each picture is indicated in the figure.

Aliquots of cells were evaluated at 24, 48, 72 and 96 hs post transfection, in order to observe cytopathic effects and viability, through counting them in a hemocytometer after the addition of 0,5% of trypan blue staining solution in optical microscopy. As negative control, cells were transfected without bacmid DNA. The transfection efficiency was measured by the percentage of blue stained cells presenting increased nuclear and membrane diameter and vacuolar structures next to the inner nuclear membrane 48–72 post transfection (Fig. 2), which are characteristic of baculoviruses infection [4], [5], [6]. Recombinant baculovirus titer was estimated by the number of blue stained cells after 48–72 post transfection associated with cytophatic effects (Fig. 2 and Table 1) and considering that each infected cell produce 100 virus particles.

## Method validation

**Fig. 3:**
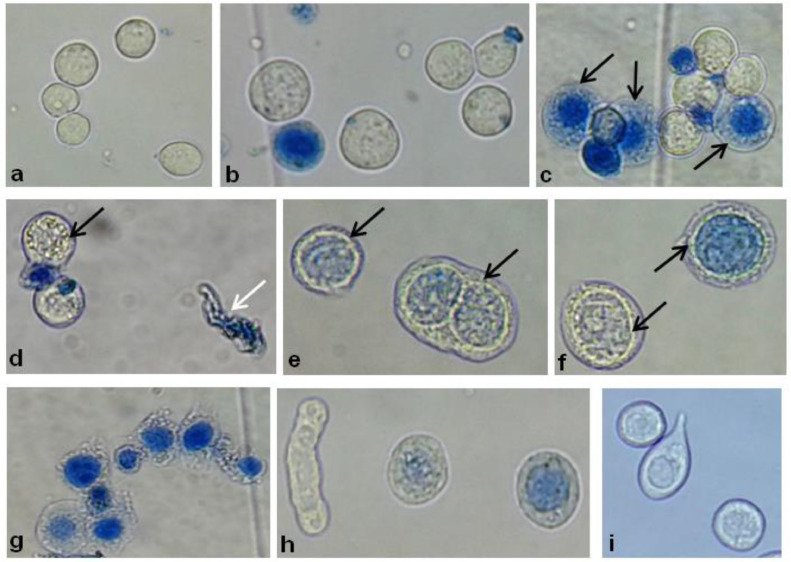
Microscopic analysis of Sf9 insect cells before and after transfection with Mayaro Nsp2 recombinant baculovirus with 40x magnification. (a) Non-infected control, (b) 24 h post transfection. (c) 48 h post transfection; black arrows show non-viable cells stained with trypan blue (d, e, f) 48 h post transfection; white arrow indicates cellular debris and the black arrow indicates the formation of vacuolar structures next to the inner nuclear membrane of the cell. (g, h, i) 72 h post transfection.

With the employment of the BEV system *Bac-to-Bac* HBM-TOPO purchased from ThermoFischer Scientific/Invitrogen, recombinant baculoviruses were obtained from ten different protein encoding genes (Table 1). Comparative features of classical [7] and modified transfection method are summarized in Table 2. The transfection efficiency of modified method varied from 45 to 57% independent from the size of the insert used in the recombinant construction. The transfection efficiency shown in Table 1 for *Leishmania* chitinase encoding gene constructs corresponds to the simple average of three different experiments, displaying the method reproducibility. Transfections of Sf9 insect cells in serum free culture medium, the ESF 921 (Expression Systems LLC) and SF900 III (Thermo Scientific/Invitrogen) presented similar results. Estimation of cytopathic effects and viability of Sf9 cells were evaluated in optical microscopy at 24, 48, 72 and 96 h post transfection, in a hemocytometer, after the addition of 0,5% of trypan blue staining solution (Fig. 2), comparatively with non-transfected cells. During the first 24 h, there was no cellular growth and the first observable cytopathic effects occurred after 48 h, corresponding to an increase in the diameter of the nuclear and plasma membranes, followed by cell unviability, observed by the incorporation of trypan blue dye (Fig. 2, 48 h). The occurrence of cell lysis was confirmed by the presence of cellular debris (Fig. 2, 96 h). Besides, evidence of baculoviruses infection was observed by vacuolar structures next to the inner nuclear membrane after 48–72 h post transfection (Figs. 3d, and 3f). Alterations in cell morphology were specific for each recombinant construction and detected 72 h post transfection (Fig. 3, g-i).

**Table 2:** Comparison of conventional and modified Sf9 transfection method.

Features	Sf9 insect cells growth condition	
	Monolayer^a^	Suspension
**Type of culture flask**	Bottle	Erlenmenyer
**Culture flask size**	75 cm^2^	25 mL
**Transfection flask size**	75 cm^2^ bottle	1.5 mL microtube
**Amount of transfection medium**	12 mL	0,7 mL
**Number of cells**	6 × 10^6^	6 × 10^6^
**Initial tranfection volume**	12 mL	12 mL
**Bacmid DNA**	6 ug	6 ug
**Cellfectin**^Ⓡ^	48 uL	8 uL
**Transfection efficiency**	2–20%	45–57%
**Virus titer (pfu/mL)**	1 × 10^6^ to 1 × 10^7^	2,2 × 10^7^ to 2,8 × 10^7^

a Data of transfection method on cells growth in monolayer were obtained from *the Bac-to-Bac*^Ⓡ^ Baculovirus Expression System manual [7].

The transfection efficiency was measured by the percentage of blue stained cells presenting increased nuclear and membrane diameter and vacuolar structures next to the inner nuclear membrane 48–72 h post transfection (Fig. 2), which are characteristic of baculoviruses infection [4], [5], [6]. Recombinant baculovirus titer was also estimated by the number of infected cells presenting specific cytopathic effect, 72 h post transfection. All constructions have an efficiency of transfection varying from 45 to 57% with an estimated initial titer of more than 2 × 10^7^ viruses/ mL and a total volume of 12 mL. The transfection efficiency of two constructions was confirmed by classical virus plate titration method, as recommended in the manufacturer manual of *Bac-to-Bac* BEVS. Using MOI of one, the first amplification of the virus titer was performed in a volume of 240 mL of Sf9 cell culture at a 1 × 10^6^ density and more than 95% of viability. The infection was observed by cytophatic effects and after 96 h, all cells were infected and the estimated titer reached than 1 × 10^8^ viruses/ mL. The second round of titer amplification was performed by infection of 100 mL of Sf9 cell culture at 1 × 10^6^ density and more than 95% of viability with 1 mL of passage 2 virus stock (MOI = 1), to obtain a virus stock of more than 1 × 10^8^ viruses / mL in 96 h. Thus, from each transfection experiment, after only two passages, it is possible to obtain 24 L of virus stock at 1 × 10^8^ viruses/ mL.

Comparatively, to obtain 24 L of 1 × 10^8^ viruses/mL after two rounds of amplification, using the classical method with a maximum of 20% of transfection efficiency, the initial transfection volume should be of 30 mL. And to obtain the same virus titer and volume, with the classical transfection method with an efficiency reaching the minimum of 2%, the initial transfection volume should be of 300 mL. Considering that conventional transfection is performed in monolayer cells grown in culture bottles, more expensive than 1,5 mL tubes, and that it is necessary to use six to twelve more *Cellfectin*^Ⓡ^, depending on the transfection efficiency obtained, the method of transfection reported here is more efficient, scalable, and present a lower cost. A higher titer of recombinant baculoviruses obtained with low passages supports production of recombinant proteins in industrial volumes, avoiding the occurrence of DIP.

## Declaration of Competing Interest

The authors declare that they have no known competing financial interests or personal relationships that could have appeared to influence the work reported in this paper.
